# Norepinephrine Drives Sleep Fragmentation Activation of Asparagine Endopeptidase, Locus Ceruleus Degeneration, and Hippocampal Amyloid-β_42_ Accumulation

**DOI:** 10.1523/JNEUROSCI.1929-23.2024

**Published:** 2024-06-03

**Authors:** Kathy Zhang, Yan Zhu, Polina Fenik, Dennis Fleysh, Colin Ly, Steven A. Thomas, Sigrid Veasey

**Affiliations:** Departments of Medicine and Pharmacology, Chronobiology and Sleep Institute, Perelman School of Medicine, University of Pennsylvania, Philadelphia, Pennsylvania 19104

**Keywords:** amyloid, hippocampus, locus ceruleus, norepinephrine, sleep deprivation, tau

## Abstract

Chronic sleep disruption (CSD), from insufficient or fragmented sleep and is an important risk factor for Alzheimer's disease (AD). Underlying mechanisms are not understood. CSD in mice results in degeneration of locus ceruleus neurons (LCn) and CA1 hippocampal neurons and increases hippocampal amyloid-β_42_ (Aβ_42_), entorhinal cortex (EC) tau phosphorylation (p-tau), and glial reactivity. LCn injury is increasingly implicated in AD pathogenesis. CSD increases NE turnover in LCn, and LCn norepinephrine (NE) metabolism activates asparagine endopeptidase (AEP), an enzyme known to cleave amyloid precursor protein (APP) and tau into neurotoxic fragments. We hypothesized that CSD would activate LCn AEP in an NE-dependent manner to induce LCn and hippocampal injury. Here, we studied LCn, hippocampal, and EC responses to CSD in mice deficient in NE [dopamine β-hydroxylase (*Dbh*)^−/−^] and control male and female mice, using a model of chronic fragmentation of sleep (CFS). Sleep was equally fragmented in *Dbh*^−/−^ and control male and female mice, yet only *Dbh*^−/−^ mice conferred resistance to CFS loss of LCn, LCn p-tau, and LCn AEP upregulation and activation as evidenced by an increase in AEP-cleaved APP and tau fragments. Absence of NE also prevented a CFS increase in hippocampal AEP-APP and Aβ_42_ but did not prevent CFS-increased AEP-tau and p-tau in the EC. Collectively, this work demonstrates AEP activation by CFS, establishes key roles for NE in both CFS degeneration of LCn neurons and CFS promotion of forebrain Aβ accumulation, and, thereby, identifies a key molecular link between CSD and specific AD neural injuries.

## Significance Statement

Sleep disruption commonly occurs and increases the risk of AD, yet molecular mechanisms are not understood. Locus ceruleus neurons (LCn) provide norepinephrine (NE) to most of the brain, where NE has largely neuroprotective roles. However, the metabolism of NE in LCn can promote the formation of pathogenic amyloid and tau fragments implicated in AD neural injury. Here, we found that sleep disruption increases the formation of toxic amyloid and tau fragments in LCn and that NE drives the formation of these fragments, LCn loss and hippocampal amyloid-β accumulation. This work identifies a molecular window into sleep loss neural injury pertinent to late-onset or spontaneous AD.

## Introduction

Chronic sleep disruption (CSD) commonly occurs through chronic short sleep or chronic fragmentation of sleep (CFS). Both forms of CSD increase the risk of developing Alzheimer's disease (AD) and related dementias (Lim et al., 2013; Sabia et al., 2021). CSD increases neuronal activity-dependent amyloid-β (Aβ) peptide and tau production, while reducing glymphatic clearance of Aβ (J. E. Kang et al., 2009; Xie et al., 2013; Holth et al., 2019). Sleep disruption in young adult wild-type mice for 1 week imparts metabolic injury to the locus ceruleus neurons (LCn; J. Zhang et al., 2014), while extended sleep disruption in young adult mice manifests as lasting neural injury, including injury to and loss of LCn and CA1 hippocampal neurons, and increased hippocampal Aβ_42_, p-tau, and glial reactivity (Owen et al., 2021), supporting the concept that CSD can influence AD-vulnerable neuronal groups and influence Aβ and tau homeostasis, irreversibly and in the absence of a genetic predisposition to AD pathology. Yet, molecular mechanisms underlying the link between sleep loss and AD are not known.

LCn are of considerable interest in AD. Across the lifespan, LCn are the earliest neurons to accumulate hyperphosphorylated tau (Braak et al., 2011), and across Braak stages of AD, independent of age, LCn are progressively lost (Theofilas et al., 2017; Oh et al., 2019). Moreover, reduced integrity of the LC in humans and poor LC responsiveness to novelty (as measured with functional imaging) predict cognitive decline and/or greater amyloid plaque and tau tangle burden (Jacobs et al., 2021; Prokopiou et al., 2022). What we cannot ascertain from observational human studies is whether injury to the LCn contributes to any of the cognitive and neuropathology findings in AD. This question has begun to be addressed in a series of AD animal model studies. Lesioning LCn axons with *N*-(2-chloroethyl)-*N*-ethyl-2-bromobenzylamine hydrochloride (DSP-4) accentuates some, but not all, features of tauopathy in the P301S mouse model (Chalermpalanupap et al., 2018). Similarly, DSP-4 increases gliosis and amyloid plaque deposition in mouse models of AD amyloid pathology (Heneka et al., 2006; Kalinin et al., 2007; Jardanhazi-Kurutz et al., 2010). These studies suggest that LCn injury may exacerbate Aβ and tau homeostasis, promoting neural injury. LCn are the primary source of norepinephrine (NE) to most areas of the brain, and NE is considered largely neuroprotective (O’Donnell et al., 2012). Thus, a question that remains unanswered following the LCn lesioning experiments is whether it is the loss of NE or axons or increased turnover of NE that contributes to the DSP-4 injury.

In support of the latter possibility, NE processing in LCn can result in the formation of toxic Aβ and tau fragments. Specifically, NE released from LCn may be taken back up into LCn varicosities or terminals and then metabolized by monoamine oxidase-A to 3,4-dihydroxyphenylglycolaldehyde (DOPEGAL), which may then activate asparagine endopeptidase (AEP) to cleave among other proteins amyloid precursor protein (APP) and tau (Zhang et al., 2015; S. S. Kang et al., 2020). The cleaved APP and tau fragments have been shown to promote Aβ_42_ production and aggregation and tau mislocalization, aggregation, and propagation (Z. Zhang et al., 2014, 2015; S. S. Kang et al., 2020, 2022).

Considering the significance of LC injury and dysfunction in predicting AD outcomes, LCn vulnerability in both CSD and in AD and the large gaps in understanding the molecular mechanisms by which sleep disruption might influence AD, we sought to gain insight into the role of NE in the neural injuries previously observed in CSD that are also observed in AD.

## Materials and Methods

### Mice and study overview

Studies were performed at the University of Pennsylvania in accordance with the National Institutes of Health Office of Laboratory Animal Welfare Policy and the Institutional Animal Care and Use Committee. Dopamine β-hydroxylase (DBH) is required for NE biosynthesis, and NE is essential for fetal survival (Thomas et al., 1995). Female and male *Dbh*^+/−^ mice with hybrid backgrounds of C57BL/6J and 129/SvCPJ.17 were mated to generate *Dbh*^−/−^, *Dbh*^+/−^, and *Dbh*^+/+^ mice. Staged dams were then treated with 100 μg/ml phenylephrine and isoproterenol in their drinking water from embryonic days 8.5–16.5 and then with 2 mg/ml ʟ-threo-3,4-dihydroxyphenylserine (ʟ-DOPS, Lundbeck Pharmaceuticals), until their pups were born. Age- and sex-matched littermate *Dbh*^+/−^ and *Dbh*^+/+^ mice were used as controls (*Dbh*^+^) for *Dbh*^−/−^ (*Dbh*^−^), as tissue content of NE and epinephrine is normal in heterozygous mice, and phenotypic differences have not been observed relative to wild-type mice (Thomas and Palmiter, 1997). Mice were exposed to a 12 h light/dark exposure and were provided food and water *ad libitum*. At 10–12 months of age, mice were randomized to 16 weeks of CFS. A subset of mice underwent sleep recording studies to assess the effectiveness of sleep fragmentation prior and again 2 months into CFS. At 16–18 months of age, 2 months after CFS, mice were perfused for immunohistologic studies.

### Sleep fragmentation protocol

CFS, which injures LCn (Li et al., 2014), was selected as our model of CSD. Mouse cages were placed atop an orbital rotor (MaxQ 2000) with the speed set at 120 rpm for 10 s/min, 24 h/d, controlled with an adjustable timer (H3CR-F8-300, Omron). Rested controls were housed in the same room without exposure to cage movement. Cages were modified with a vertical extender to allow water bottles with an elongated nozzle and ball valve to prevent water leakage. This method of sleep fragmentation does not elevate plasma corticosterone levels; mice maintain body weight, and the frequency of arousals in wild-type mice remains elevated across weeks of exposure (Li et al., 2014).

### Electrode implantation and sleep recording and analysis

*Dbh*^−^ and *Dbh*^+^ mice were implanted with electroencephalographic (EEG) and electromyographic (EMG) electrodes for recording behavioral states. General anesthesia was induced with 3–4% isoflurane via mask and then maintained with 1–2.5% isoflurane. Using sterile procedures, two silver EEG electrodes (787000, A-M Systems) were placed bilaterally above the dorsal hippocampus, and reference electrodes were implanted over the rostral skull. Nuchal EMG electrodes were embedded in the dorsal nuchal musculature. Implanted electrodes were attached to a connector pedestal (MS363, Plastics One), which was secured with dental acrylic (C&B Metabond, Parkell). Mice recovered with littermates for 10 d and were then placed in individual cages for 3 d, prior to connecting the recording cable to a commutator (363 SL/6, SL6C Plastics One). Recordings were obtained under rested and CFS conditions at Week 8 CFS (*n* = 4–5/genotype). EEG and EMG signals were acquired at 256 Hz sampling frequency and amplified and filtered (EEG, 0.5–30 Hz and EMG, 1–100 Hz; 15A94 Grass Technologies). Data were recorded on AcqKnowledge3 software and analyzed as 4 s epochs in SleepSign (3.2, Kissei Comtec). Each epoch was scored as wake, nonrapid eye movement (NREM) sleep, or rapid eye movement (REM) sleep. Arousals were defined as the occurrence of one or more wake consecutive epochs following five or more consecutive sleep epochs (NREMS and/or REMS). The number of arousals/hour of sleep within the 24 h period is termed the arousal index, and this measure was used to characterize sleep fragmentation. Wake and NREMS bout numbers and durations were used as a secondary measure of sleep fragmentation. Because sleep has been described previously in *Dbh*^−^ mice, additional sleep analyses were limited to pertinent outcomes: the arousal index, bout lengths and durations, and the percentage time in each stage/24 h, the latter to exclude confounds of chronic short sleep.

### Histology, microscopy, and stereology

To examine the role NE plays in LCn, CA1, and EC responses to CFS, age- and sex-matched *Dbh*^+^ and *Dbh*^−^ mice exposed to rested or CFS conditions were transcardially perfused with 4% paraformaldehyde. Brains were postfixed overnight, cryopreserved, and then sectioned in a 1:6 series of 60 μm free-floating sections for specific immunohistological responses to sleep and genotype conditions. Details of the assessed primary antibodies for immunohistology are presented in Table 1.

**Table 1.** Source and concentration details of primary antibodies used in experiments

Primary antibodies	Catalog number/company	IHC dilution
Aβ_42_	Ab120959/Abcam	1:500
AEP	PA581435/Fisher	1:400
AEP-cleaved (N373) APP	ABN 1643/Millipore Sigma	1:500
AEP-cleaved (N368) Tau	ABN1703/Millipore Sigma	1:500
CD68	Ab125212/Abcam	1:400
p-tau (S202/T205) AT8	MN1020/Invitrogen	1:500
Iba-1	Ab5076/Abcam	1:1,000
Tau-5	AH0042/Thermo Fisher Scientific	1:500
TH	AB152/Millipore Sigma	1:2,000
TH	NB 300-110/Novus Biologicals	1:2,000

We examined how NE influences the LCn survival response to CFS by carrying out stereology on Vector blue substrate (peroxidase-based, VectorLabs) labeled tyrosine hydroxylase (TH) positive neurons in pontine–caudal midbrain brain sections with Giemsa counter-staining. Five sections each spaced 120 μm and spanning bregma AP −5.20 to −5.65 mm (to represent the full rostral caudal extent of the LC nucleus) and matched across all mice were selected for *n* = 5–6 mice/sleep and genotype conditions. A Leica DM4B microscope equipped with a Stereo Investigator workstation (MicroBrightField) was used following an optical fractionator strategy. Our sampling scheme used a 0.25 *x*, *y* sampling frequency with a *z*-depth sampling of 0.80 to allow 2 μm guard zones for irregular surfaces and surface differences in immunolabeling on both sides. Previously, we validated this strategy and confirmed >150 counts/healthy adult mouse and Gundersen coefficients of error <0.10 (J. Zhang et al., 2014). Counters were blinded to genotype and sleep conditions. Giemsa-labeled nuclei within TH-labeled somata with clear Giemsa labeling of nuclear chromatin that came into focus within the *x*, *y*, and *z*-counting frame were counted in each section/mouse using a 100× magnification oil objective to calculate LCn counts/mouse in the software.

LC, CA1, and/or EC were then examined for genotype and sleep condition influences on asparagine endopeptidase (AEP), tau and APP products from AEP cleavage, phosphorylation of tau (p-tau), Aβ_42_, and microglia (Iba-1, CD68). One to three sections/mouse (*n* = 6–11 mice per genotype/sleep group) were processed with primary antibodies listed in Table 1 and appropriate secondary donkey anti-goat, anti-mouse, anti-rabbit, or anti-sheep antibodies labeled with Alexa Fluor 488, 594, or 647. Confocal microscopy (Leica SP5 AOBS) was used to image sections where laser intensity, exposure time, detector gain, amplifier offset, and depth of the focal plane within the section were standardized across compared image acquisitions. All image analyses were performed with scorers blinded to conditions using NIH ImageJ software by converting images to grayscale and measuring mean gray value over individual LCn (AEP, AEP-tau, AEP-APP) or by converting to 8 bit grayscale, inverting and measuring percentage area within a region of interest and using a standardized threshold for the image set. Immunofluorescent mean gray data were normalized to rested *Dbh*^+^ values.

### Statistical analysis

Statistical analyses were performed using GraphPad statistical software (Prism, versions 6.0 and 11.2). To examine how preselected behavioral state parameters varied with sleep conditions and genotype, a repeated measures analysis (within animal rested and CFS) was implemented. Analyses included assessment of an interaction effect between sleep condition and genotype (sleep × genotype) and/or main effects of sleep condition and genotype. Data were then compared across the sleep conditions and genotypes to determine the significance of group differences. To examine the effects of sleep condition and genotype on histopathologic variables, two-way ANOVA (full model with sleep condition, genotype, and sleep × genotype interaction) was used, with Tukey's multiple-comparisons post hoc (*q*) analyses. The cutoff for significant statistical power for all analyses was a multiple-comparisons corrected *p* value <0.05.

## Results

### Rotor platform motion fragments sleep in *Dbh*^+^ and *Dbh*^−^ mice

**Figure 1.**
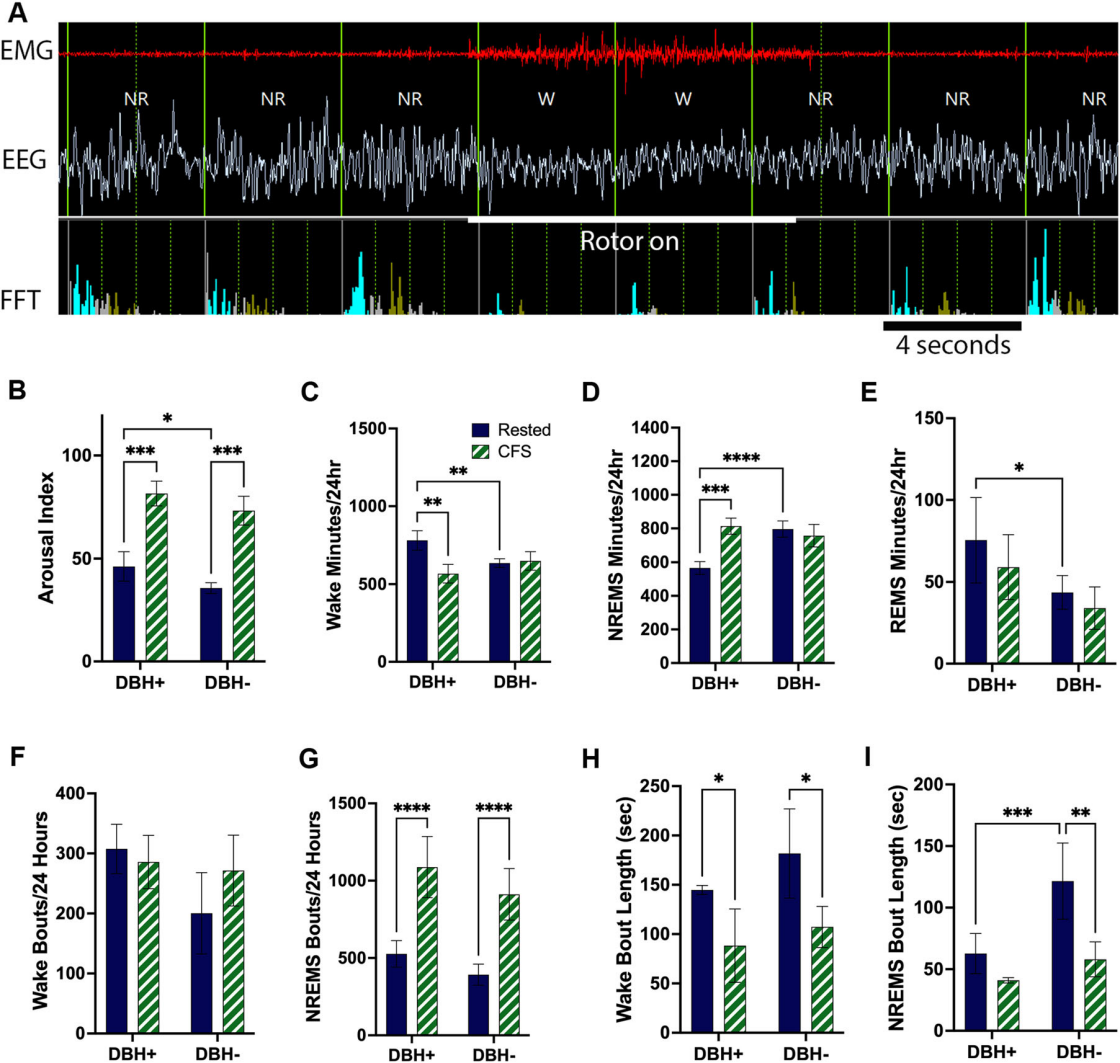
Rotor platform rotation comparably fragments sleep in *Dbh*^+^ and *Dbh*^−^ mice. ***A***, A representative polygraphic raw data image of an elicited arousal from nonrapid eye movement (NR) sleep. Top red tracing depicts the electromyographic (EMG) signal, and the white signal at the bottom shows the electroencephalographic (EEG) signal. The bottom panel bins EEG fast Fourier transform (FFT) power bins with blue representing delta or 0–4 Hz, gray for theta 6–9 Hz, and green for alpha, 11–14 Hz. The large white line marks the 10 s when the rotor platform is moving. The timing calibration bar is 4 s. ***B***, Arousal index (number of arousals from sleep/total sleep time in hours) across two genotypes (*Dbh*^+^ and *Dbh*^−^) for two sleep conditions, rested, navy and chronic fragmentation of sleep, CFS, green/white). Shown are mean ± SE for *n* = 4/group. ***C–E***, Twenty-four hour time (minutes) spent in each of the three behavioral states: wake (***C***), nonrapid eye movement sleep (NREMS, ***D***), and rapid eye movement sleep (REMS, ***E***) for *Dbh*^+^ and *Dbh*^−^ mice (*n* = 4/group, males and females balanced) analyzed with repeated measures two-way ANOVA and Tukey's post hoc testing. ***F***, ***G***, Number of wake (***F***) and NREMS (***G***) bouts/24 h in the same mice as for arousal index and behavioral state times. Values are mean ± SE. ***H***, ***I***, Duration of wake (***H***) and NREMS bout durations, as mean ± SE. In all graphs, navy represents baseline (rested) conditions and green/white diagonals represent CFS conditions. *p < 0.5; **p < 0.01; ***p < 0.001; and ****p < 0.0001.

Sleep fragmentation is defined as an increase in the frequency of arousals from sleep (NREM and REM sleep), and this measure is termed the arousal index. A typical example of rotor-elicited arousal from NREMS is provided in Figure 1*A*. Elicited arousals appeared similar for the two genotypes. Arousal indices were compared across the two genotypes and two sleep conditions (rested and CFS). Although there was no sleep × genotype interaction for arousal index, there were both genotype, *F*_(1,6)_ = 11; *p* < 0.05, and sleep, *F*_(1,6)_ = 133; *p* < 0.0001 main effects. Specifically, the arousal index across rested conditions was slightly higher in *Dbh*^+^ than that in *Dbh*^−^ mice (*t* = 2.5; *p* < 0.05). For both genotypes, CFS increased the arousal index (*Dbh*^+^, *t* = 7.9; *p* < 0.001 and *Dbh*^−^, *t* = 8.4; *p* < 0.001), so that in response to CFS, the arousal index was similar for CFS *Dbh*^+^ and *Dbh*^−^ mice (*t* = 2.0, N.S.), as summarized in Figure 1*B*. The effects of NE deficiency on behavioral states have been described, where overall times in wake and REM sleep are reduced across the 24 h light/dark cycle and NREM sleep time is increased (Ouyang et al., 2004). We next analyzed total behavioral state times/24 h for each of the states before and during CFS in the two strains. For wakefulness, there was a sleep and genotype interaction, *F*_(1,6)_ = 19; *p* < 0.01, and a main sleep effect (*F*_(1,6)_ = 14; *p* < 0.01). Specifically, wake time for 24 h was 140 min higher in rested *Dbh*^+^ when compared with rested *Dbh*^−^ mice (*t* = 3.8; *p* < 0.01; Fig. 1*C*). CFS resulted in 200 fewer minutes of wake time in *Dbh*^+^ mice, relative to their rested wake time (*t* = 5.8; *p* < 0.01), while CFS did not affect wake time in *Dbh*^−^ mice (*t* = 0.4, N.S.). These responses contributed toward similar wake/24 h times in CFS *Dbh*^+^ and CFS *Dbh*^+^ mice (*t* = 2.1, N.S.). For NREMS (Fig. 1*D*), a sleep × genotype interaction was also evident, *F*_(1,6)_ = 40; *p* < 0.001, and both sleep and genotype main effects were observed, *F*_(1,6)_ = 9.2; *p* < 0.01 and *F*_(1,6) _= 21.0; *p* < 0.05, respectively. Rested *Dbh*^+^ mice had 230 fewer minutes of NREM sleep than rested *Dbh*^−^ mice (*t* = 6.3; *p* < 0.0001). NREMS time increased in response to CFS for *Dbh*^+^ mice; *t* = 7.7; *p* < 0.001), without changing in *Dbh*^−^ mice (*t* = 1.2, N.S.) so that NREMS times, like wake times, did not differ between CFS *Dbh*^+^ and CFS *Dbh*^−^ mice (*t* = 1.6, N.S.). For REM sleep (Fig. 1*E*), the only main effect observed was in genotype, *F*_(1,6)_ = 6.9; *p* < 0.05, where rested *Dbh*^+^ mice had 30 min more REMS than *Dbh*^−^ mice, *t* = 2.5; *p* < 0.05. During CFS, there were no genotype differences for REMS (*t* = 1.9, N.S.). To further characterize disruption of sleep in our paradigm in the two strains, we assessed the effects of CFS on wake and NREMS bout numbers and bout durations. Overall, for wake bout numbers/24 h, there were no sleep × genotype interaction, *F*_(1,6)_ = 4, N.S. and no main effect for sleep or genotype, *F*_(1,6)_ = 1 and 4, respectively, N.S., as summarized in Figure 1*F*. For NREMS bout numbers, a main effect for sleep condition was observed, *F*_(1,6)_ = 136; *p* < 0.0001, where bout numbers increased in response to CFS in both genotypes (both *t* = 11.3; *p* < 0.0001; Fig. 1*G*). For the mean duration of wakefulness bouts (Fig. 1*H*), there was no sleep × genotype effect, *F*_(1,6)_ = 0, N.S., and no effect of genotype on duration of wake bouts, *F*_(1,6)_ = 3, N.S., yet the mean duration of wake bouts was affected by sleep condition, *F*_(1,6)_ = 19; *p* < 0.01, where both *Dbh*^+^ and *Dbh*^−^ genotypes showed shorter durations of wakefulness after CFS, *t* = 2.7; *p* < 0.05 and *t* = 3.5; *p* < 0.05, respectively. For mean duration of NREMS bouts (Fig. 1*I*), overall effects were identified for sleep × genotype *F*_(1,6)_ = 7; *p* < 0.05, and for both sleep, *F*_(1,6)_ = 27; *p* < 0.01, and genotype, *F*_(1,6)_ = 13; *p* < 0.05. Specifically, NREMS bout lengths were shorter in rested *Dbh*^+^ mice relative to rested *Dbh*^−^ mice, *t* = 4.3; *p* < 0.001. In response to CFS, NREMS bout lengths did not change appreciably in *Dbh*^+^ mice, *t* = 0.1, N.S., yet NREMS bouts were shorter in CFS-exposed *Dbh*^−^ mice, relative to rested *Dbh*^−^ mice, *t* = 5.5; *p* < 0.01. Thus, CFS effectively disrupts sleep continuity comparably in both genotypes, and under conditions of CFS, wake and NREMS bout numbers and durations and the time/day spent in each behavioral state were similar for the two genotypes.

### CFS upregulates and activates AEP in LCn in an NE-dependent manner

**Figure 2.**
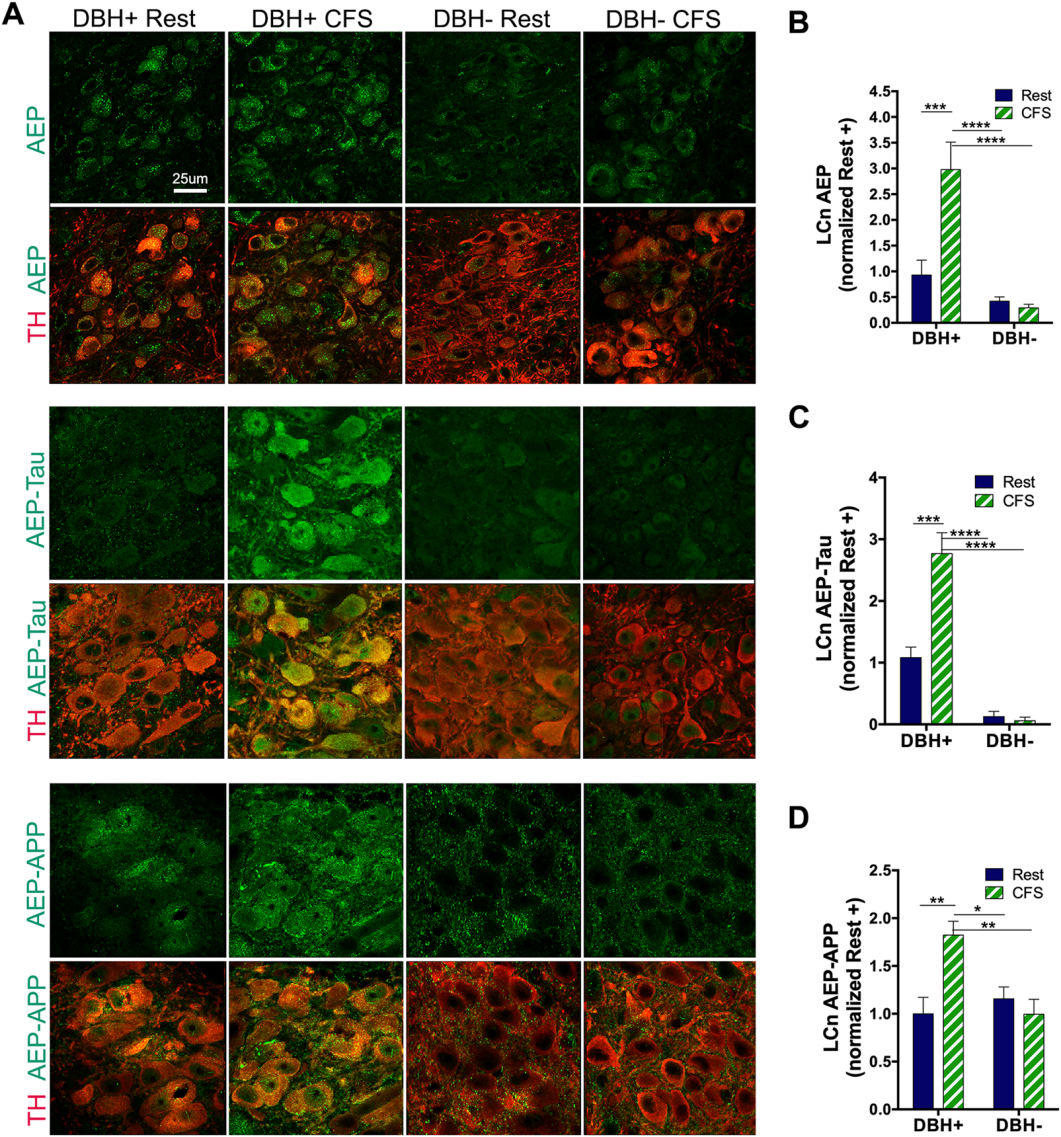
CFS upregulates and activates AEP in LCn in an NE-dependent fashion. ***A***, Representative confocal images are presented for LCn responses across sleep and genotype conditions for AEP (green, top panels), AEP-tau (green, middle panels), and AEP-APP (green, bottom panels) with and without TH-labeled LCn (red). Scale bar, 25 μm. ***B–D***, Data are summarized for mean gray area SE ± normalized to mean rested *Dbh*^+^ values in *n* = 7–11 (male and female balanced) mice/group for AEP (***B***), AEP-tau (***C***), and AEP-APP (***D***), analyzed with two-way ANOVA and Sidak's post hoc analyses. Significant differences are denoted as *p < 0.05; **p < 0.01; ***p < 0.001; ****p < 0.0001.

LCn in *Dbh*^+^ and *Dbh*^−^ mice exposed to rest or CFS conditions for 16 weeks were examined for AEP responses to CFS. Two sections/mouse (*n* = 7–11 mice/group) were analyzed. Males and females were balanced and combined in analysis. There were main genotype, *F*_(1,31)_ = 13.4; *p* < 0.0001, and sleep condition effects, *F*_(1,31)_ = 4.5; *p* < 0.05, on LCn AEP and an overall interaction *F*_(1,31)_ = 5.9; *p* < 0.05. Specifically, *Dbh*^+^ CFS mice had increased AEP relative to the other three groups: rested *Dbh*^+^ mice (*q* = 4.7; *p* < 0.05), *Dbh*^−^ CFS mice (*q* = 6.2; *p* < 0.001), and rested *Dbh*^−^ mice (*q* = 5.8; *p* < 0.01). Figure 2, *A* and *B*, shows representative images of LCn AEP across the four groups and group results. AEP cleaves tau at N255 and N368 (Z. Zhang et al., 2014) and cleaves APP at N373 and N585, where these particular cleavage sites are specific to AEP (Zhang et al., 2015). Therefore, to examine AEP activation, we assessed LCn for the presence of two AEP-cleaved products: AEP-tau_368_ (AEP-tau) and AEP-APP_373_ (AEP-APP). There were main genotype and sleep condition effects on LCn AEP-tau, *F*_(1,25)_ = 49.1; *p* < 0.0001 and *F*_(1,25)_ = 9.5; *p* < 0.01, respectively, and a sleep × genotype interaction, *F*_(1,25)_ = 11.2; *p* < 0.01 (Fig. 2*A*,*C*). In parallel with AEP results, AEP-tau was increased in CFS *Dbh*^+^ mice relative to rested *Dbh*^+^ mice (*q* = 7.0; *p* < 0.01), CFS *Dbh*^−^ mice (*q* = 10.8; *p* < 0.0001), and rested *Dbh*^−^ mice (*q* = 10.5; *p* < 0.0001). Like LCn AEP-tau, LCn AEP-APP was affected by both genotype and sleep conditions, *F*_(1,33)_ = 4.9; *p* < 0.05 and *F*_(1,33)_ = 4.8; *p* < 0.05, respectively, and an overall interaction was observed, *F*_(1,33)_ = 10.6; *p* < 0.01 (Fig. 2*A*,*D*). Post hoc Tukey's test found that CFS *Dbh*^+^ mice had increased LCn AEP-APP, relative to the other three groups: rested *Dbh*^+^ mice (*q* = 5.5; *p* < 0.01), CFS *Dbh*^−^ mice (*q* = 5.4; *p* < 0.01), and rested *Dbh*^−^ mice (*q* = 4.3; *p* < 0.05). In summary, in LCn, CFS upregulates AEP and at least two of its cleavage products, AEP-APP and AEP-tau, supporting AEP activation and the ability for CFS to upregulate in LCn specific toxic APP and tau fragments known to promote Aβ aggregation and tau aggregation and propagation.

### CFS increases tau phosphorylation in LCn in an NE-dependent manner and without increasing total tau

**Figure 3.**
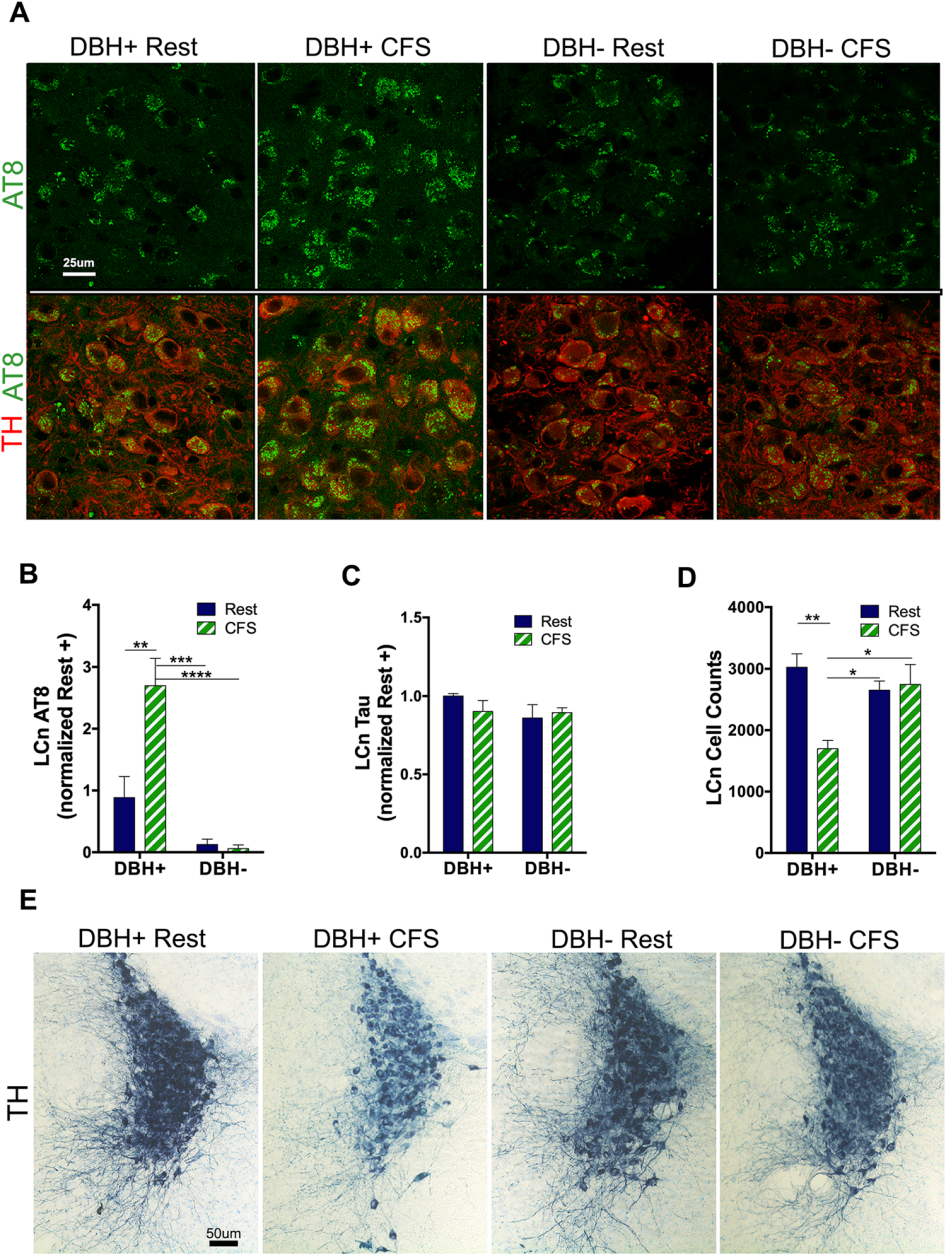
The CFS increase in p-tau in LCn and LCn loss require NE. ***A***, LCn confocal images of p-tau (P Ser202/Thr205, AT8, green) in TH (red)-labeled LCn neurons across groups. Scale bar, 25 μm. ***B***, ***C***, Summary data (mean ± SE) for LCn AT8 (***B***) and LCn total tau (***C***) for mean gray normalized to mean for rested *Dbh*^+^ mice (*n* = 6–8/group, M&F) analyzed with two-way ANOVA and Sidak's: *p* < 0.01; ***p < 0.001; ****p < 0.0001. ***D***, Optical fractionator stereological estimates of LCn counts bilaterally for the four groups, mean ± SE, *n* = 4–6/group, analyzed two-way ANOVA and Sidak's: *p < 0.05; **p < 0.01. ***E***, Representative coronal images of mid-LC region TH immunopositive (navy, substrate blue-labeled) neurons and dendritic field across the four conditions. Sections are counter-stained with Giemsa to delineate nuclei. Scale bar, 50 μm.

Across the lifespan, there is a progressive increase in brain tau phosphorylation (p-tau) at Ser-202 and Thr-205 in humans, detected with AT8 antibody, where deposition is first observed in LCn (Braak et al., 2011). Mechanisms by which this occurs are not known, but AT8 immunoreactivity in LCn predicts overall cognitive impairment across individuals with mild cognitive impairment and AD (Grudzien et al., 2007). AEP activation has been shown to increase p-tau including at Thr-205 (Basurto-Islas et al., 2013). In murine models of amyloid pathology and tauopathy, AT8 immunoreactivity increases in parallel with increases in AEP-tau cleaved at N368 (S. S. Kang et al., 2020). As observed above for AEP, a significant sleep × genotype interaction was observed for AT8, *F*_(1,24)_ = 6.6; *p* < 0.05, along with main genotype, *F*_(1,24)_ = 21.5; *p* < 0.0001, and sleep effects, *F*_(1,24)_ = 5.7; *p* < 0.05 (Fig. 3*A*,*B*), where AT8 immunoreactivity was increased in LCn in CFS *Dbh*^+^, relative to rested *Dbh*^+^ mice, *q* = 5.2; *p* < 0.01, while no increase was observed for CFS *Dbh*^−^ relative to rested *Dbh*^−^ mice, *q* = 0.2, N.S. Consequently, LCn AT8 was greater in CFS *Dbh*^+^ mice, relative to CFS *Dbh*^−^ mice, *q* = 7.6; *p* < 0.0001; Figure 3*A*,*B*. We next explored whether total tau was upregulated in LCn somata to explain increased AT8. The Tau-5 antibody epitope targets a mid-tau region devoid of phosphorylation sites, allowing detection of both phosphorylated and nonphosphorylated (total) tau. There were no genotype/sleep interactions, *F*_(1,22)_ = 1.7, N.S., and no genotype or sleep main effects, *F*_(1,22)_ = 1.3, N.S.; *F*_(1,22)_ = 0.3, N.S., respectively (Fig. 3*C*). In summary, CFS increases phosphorylation of tau in LCn at Ser202/Thr205 in an NE-dependent manner.

### CFS loss of LCn is dependent on the presence of NE

Optical fractionator stereological counts were performed across the four groups of mice (*n* = 5–6/group) to assess genotype and CFS effects on LCn counts. An overall interaction was observed, *F*_(1,18)_ = 11.5; *p* < 0.01, and while a main genotype effect was not observed, *F*_(1,18)_ = 2.5, N.S., a main sleep effect was observed, *F*_(1,18)_ = 8.7; *p* < 0.01. CFS reduced LCn counts in *Dbh*^+^, *q* = 6.4; *p* < 0.01, while CFS had no effect on LCn counts in *Dbh*^−^ mice, *q* = 0.4; N.S., Figure 3*D*,*E*. LCn counts in CFS *Dbh*^+^ mice were lower than counts in both rested and CFS *Dbh*^−^ mice (*q* = 4.8; *p* < 0.05 and *q* = 5.0; *p* < 0.05). Thus, NE is necessary for the loss of LCn in response to CFS.

### CFS- and NE-dependent AEP responses are evident in HC CA1

**Figure 4.**
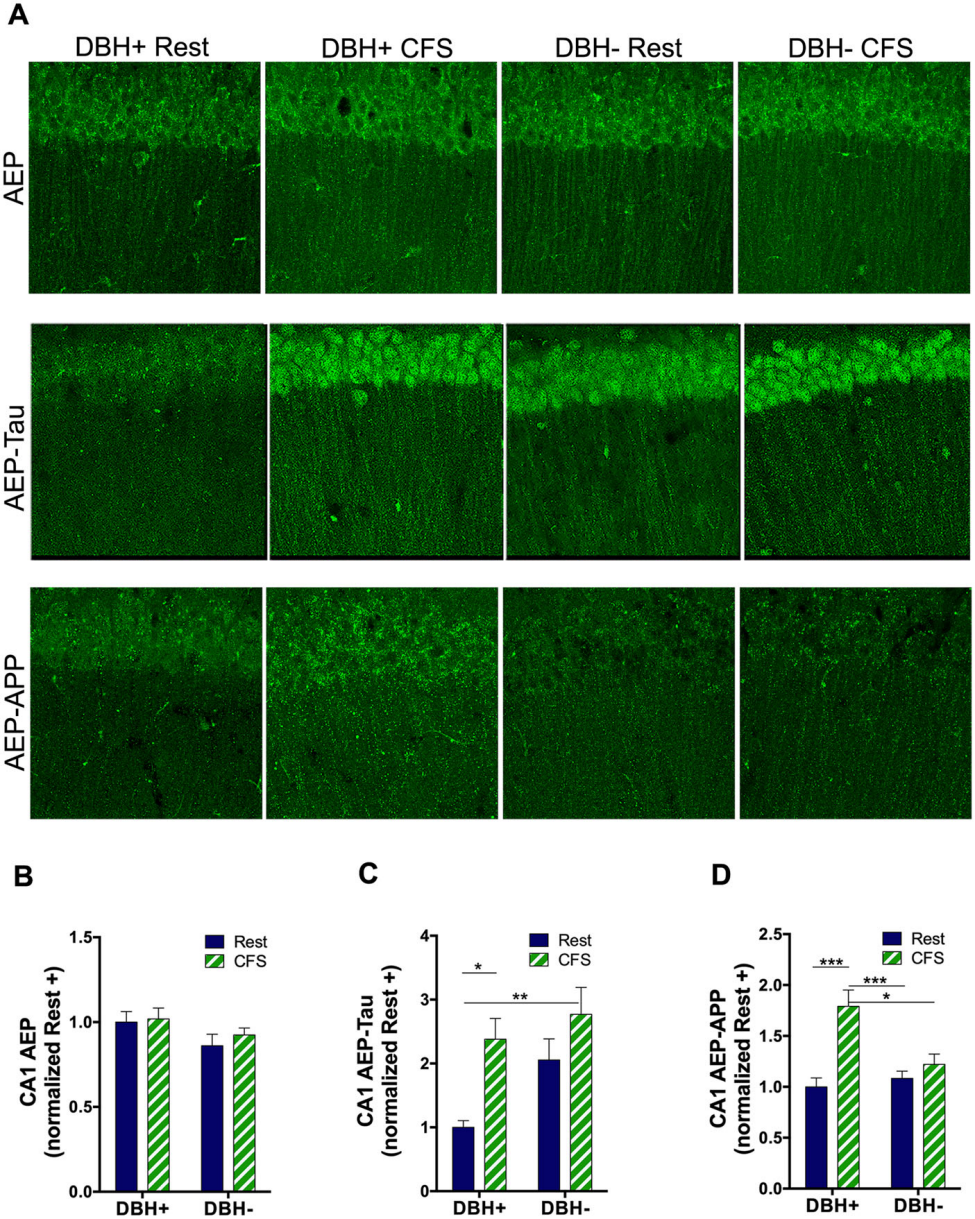
CA1 hippocampal AEP responses to CFS show NE-dependent and -independent effects. ***A***, Confocal images in CA1 of pyramidal cell layer and stratum radiatum across groups showing AEP (green, top panels,), AEP-tau (green, middle panels), and AEP-APP (green, bottom panels), Scale bar, 100 μm. ***B–D***, Group data (mean + SE, normalized to mean of rested *Dbh*^+^ for mean gray area, *n* = 5–7) in CA1 for AEP (***B***), AEP-tau (***C***), and AEP-APP (***D***) for rested (navy) and CFS (green/white), analyzed with two-way ANOVA and Tukey's post hoc analyses. Significant differences are denoted as *p < 0.05; **p < 0.01; ***p < 0.001.

To ascertain how generalizable the CFS-induced increase in AEP is and its activation, we next examined AEP responses in the CA1 region of the hippocampus. In contrast with LCn genotype/sleep effects, there was no overall interaction, *F*_(1,29)_ = 0.1, N.S., and no main genotype or sleep effect for CA1 AEP, *F*_(1,29)_ = 3.5, N.S. and *F*_(1,29)_ = 0.4, N.S., respectively (Fig. 4*A*,*B*). AEP, however, may be activated in the absence of protein upregulation by the presence of oxidative stress and/or an acidic environment (Zhao et al., 2014). To assess CFS effects on AEP activity, we next examined the effects of sleep disruption and *Dbh* genotype on AEP-tau in CA1. Both genotype and sleep main effects were observed (*F*_(1,32)_ = 5.6; *p* < 0.05 and *F*_(1,32)_ = 11.3; *p* < 0.01) without a sleep × genotype interaction (*F*_(1,32)_ = 1.2, N.S.). Post hoc analyses revealed an increase in *Dbh*^+^ mice from rested to CFS, *q* = 4.7; *p* < 0.05. AEP-tau was higher in CFS *Dbh*^−^ mice than in rested *Dbh*^+^ mice, *q* = 5.8; *p* < 0.05; *p* < 0.01 (Fig. 4*A*,*C*). For CA1 AEP-APP, a genotype/sleep interaction was found (*F*_(1,30)_ = 6.5; *p* < 0.05) and a main sleep effect was observed (*F*_(1,30)_ = 17.5; *p* < 0.001) without an overall genotype effect (*F*_(1,30)_ = 2.9, N.S.). Specifically, AEP-APP was higher in CFS *Dbh*^+^ mice than that in any of the other groups: rested *Dbh*^+^ (*q* = 7.0; *p* < 0.001), rested *Dbh*^−^ (*q* = 6.1; *p* < 0.001) and CFS *Dbh*^−^ mice (*q* = 4.1; *p* < 0.05; Fig. 4*A*,*D*). In summary, without a measurable increase in AEP, CFS activates AEP in the HC, increasing both AEP-tau and AEP-APP in wild-type mice. The absence of an increase in AEP supports metabolic AEP activation in LCn by CFS. That *Dbh*^−^ mice show increased AEP-tau in rested conditions suggests that AEP targeting of APP occurs independently from AEP targeting of tau in the HC, suggesting differences in subcellular localization.

### CFS- and NE-dependent AEP responses in EC are consistent with CA1 responses

**Figure 5.**
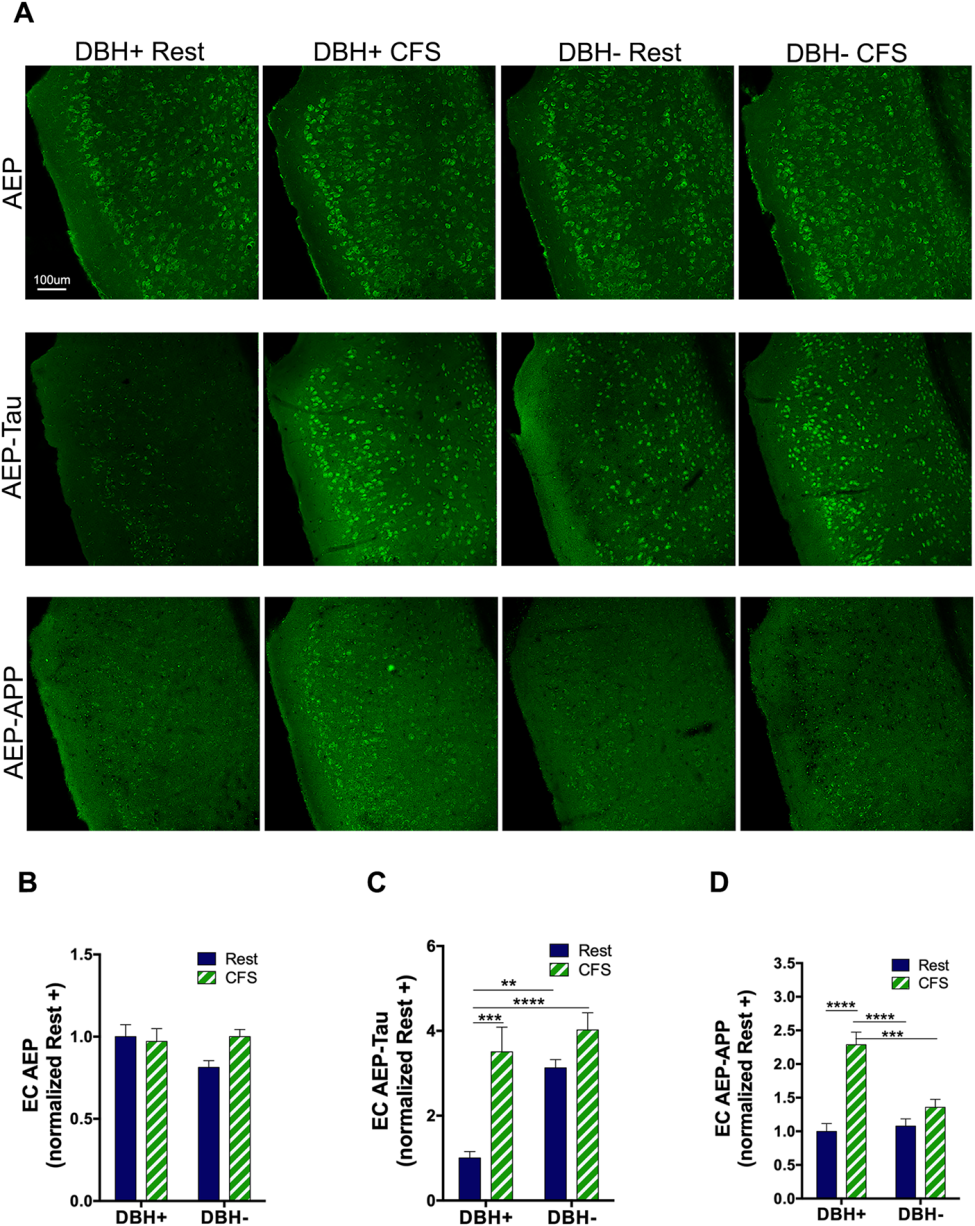
NE-dependent and NE-independent effects for EC AEP responses to CFS. ***A***, Confocal images in EC (layers I–VI) across the four conditions showing AEP (green, top panels), AEP-tau (green, middle panels), and AEP-APP (green, bottom panels). ***B–D***, Group data (mean + SE, normalized to mean of rested *Dbh*^+^ for mean gray area, *n* = 5–7) in EC for AEP (***B***), AEP-tau (***C***), and AEP-APP (***D***) for rested (navy) and CFS (green/white), analyzed with two-way ANOVA and Sidak's post hoc analyses. Significant differences are denoted as **p < 0.01; ***p < 0.001; ****p < 0.0001.

As observed in CA1, in the EC, a sleep × genotype interaction was not present for AEP, *F*_(1,29)_ = 4.1, N.S., and there were no main genotype or sleep effects, *F*_(1,29)_ = 0.9, N.S. and *F*_(1,29)_ = 0.9, N.S., respectively (Fig. 5*A*,*B*). As observed in CA1, main sleep condition and genotype effects were evident for AEP-tau in EC, *F*_(1,24)_ = 21.3; *p* < 0.0001, and *F*_(1,24)_ = 21.4; *p* < 0.0001, respectively, while no sleep × genotype interaction was evident, *F*_(1,24)_ = 3.1; N.S. Specifically, AEP-tau in rested *Dbh*^+^ was lower relative to CFS *Dbh*^+^ mice, *q* = 6.9; *p* < 0.001; rested *Dbh*^−^, *q* = 6.2; *p* < 0.01; and CFS *Dbh*^−^ mice, *q* = 9.0; *p* < 0.0001, as shown in Figure 5*A*,*C*. Main genotype and sleep condition differences were observed for AEP-APP in the EC (*F*_(1,30)_ = 8.3; *p* < 0.01 and *F*_(1,30)_ = 30.8; *p* < 0.0001, respectively), and a sleep × genotype interaction was present (*F*_(1,30)_ = 12.6; *p* < 0.01; Fig. 5*A*,*D*). As seen in CA1, post hoc comparisons of AEP-APP in EC revealed increased EC AEP-APP in CFS *Dbh*^+^ relative to the other three groups: rested *Dbh*^+^, *q* = 9.7; *p* < 0.0001; rested *Dbh*^−^, *q* = 8.7; *p* < 0.0001; and CFS *Dbh*^−^, *q* = 6.4; *p* < 0.001. In summary, responses for AEP, AEP-tau, and AEP-APP in the EC mirrored responses for CA1 and both suggest sleep and genotype divergences in AEP targeting of tau and APP in the forebrain.

### Aβ_42_ and tau phosphorylation responses are influenced by CFS and/or NE in CA1 and EC

**Figure 6.**
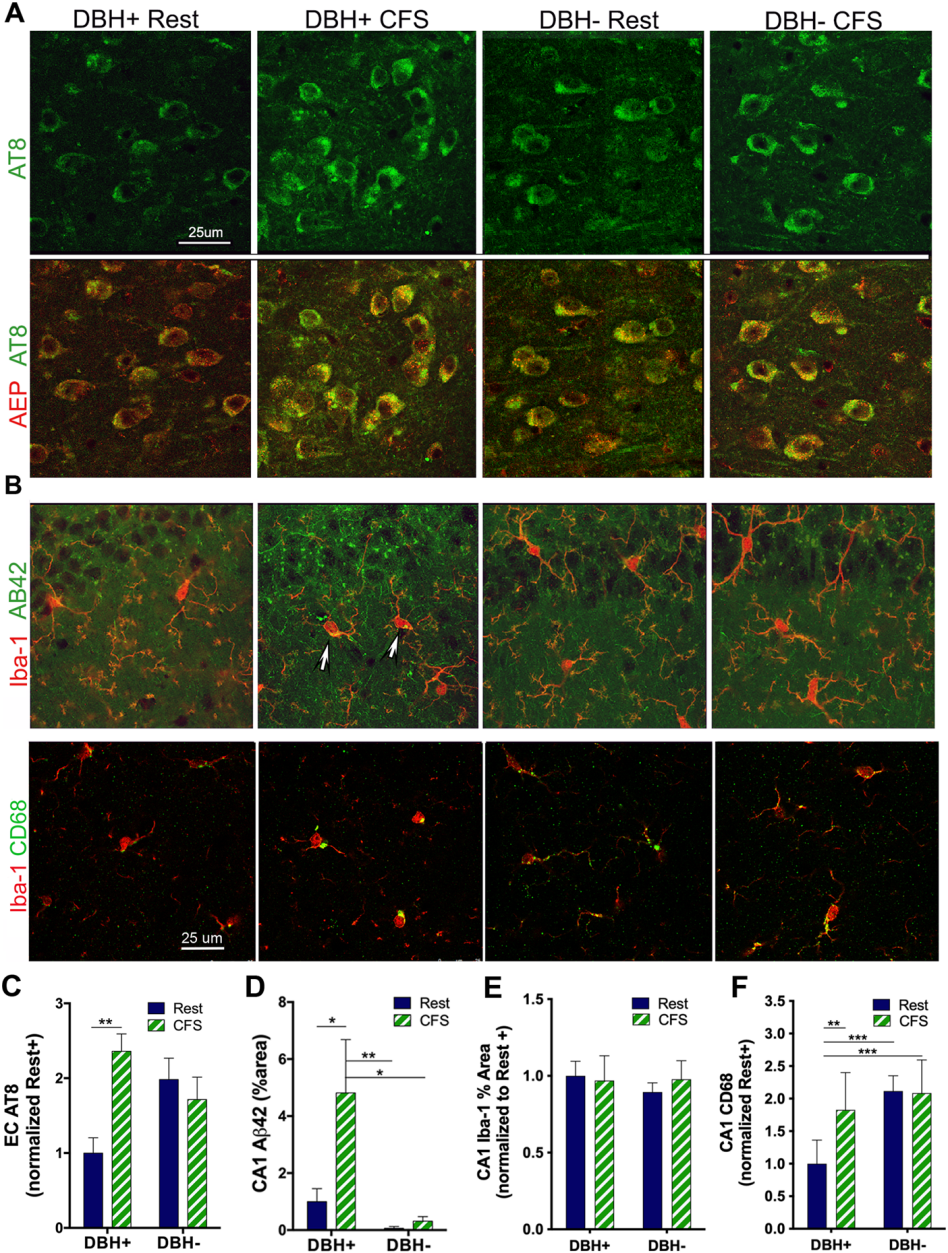
Effects of CFS and NE deficiency on p-tau in EC and Aβ_42_ in CA1. ***A***, Representative confocal images of p-tau (AT8) (green) and AEP (red) within the EC. Scale bar, 25 μm. ***B***, Confocal images of Aβ_42_ (green) and Iba-1 (red) in CA1 at pyramidal cells and dorsal stratum radiatum across groups (top panel). Arrows highlight colocalization of Aβ_42_ in Iba-1 + cells and CD68 (green) and Iba-1 (red) images across groups, bottom panel. Scale bar, 25 μm. ***C–F***, Group data (mean + SE; *n* = 6–8) in EC for AT8 (***C***), normalized to mean of rested *Dbh*^+^ for mean gray area, for Aβ_42_ in CA1 (***D***) expressed as percent area above set threshold; for CA1 Iba-1 (***E***), expressed as % area above set threshold; and CD68 (***F***) normalized to rested *Dbh*^+^ for rested (navy) and CFS (green/white), analyzed with two-way ANOVA and Sidak's post hoc analyses. Significant differences are denoted as *p < 0.05; **p < 0.01; ***p < 0.001.

We have shown that chronic short sleep increases Aβ_42_ in CA1 and increases p-tau (Ser202/Thr205, AT8) in both CA1 and the EC in wild-type mice (Owen et al., 2021). Here, we examined whether CFS influences p-tau and Aβ_42_ in wild-type mice and whether NE influences the Aβ and p-tau responses to sleep disruption. A strong AT8 signal was evident in the EC, with negligible signal in the CA1 region, and thus measurements were obtained only in the EC. AT8 was measured in the EC across layers II–VI, and while a main genotype effect was not observed, *F*_(1,19)_ = 0.5, N.S., a main sleep effect, *F*_(1,19)_ = 4.5; *p* < 0.05, and a sleep × genotype interaction, *F*_(1,19)_ = 10.0; *p* < 0.01, were observed. CFS increased EC AT8 in *Dbh*^+^ mice relative to rested *Dbh*^+^ mice, *q* = 5.4; *p* < 0.01 (Fig. 6*A*,*C*). There were no significant differences in AT8 across the other groups (*q* = 1.7–3.0, N.S.). A detectable Aβ_42_ signal was evident in CA1 without signal detected in the EC; thus, Aβ_42_ measurements were obtained only in CA1. Main genotype and sleep effects were seen for CA1 Aβ_42_ (*F*_(1,36)_ = 8.8; *p* < 0.01 and *F*_(1,36)_ = 4.8; *p* < 0.05), without a sleep × genotype interaction *F*_(1,36)_ = 3.0, N.S. CA1 Aβ_42_ was increased in CFS *Dbh*^+^ mice, relative to the other three groups: rested *Dbh*^+^, *q* = 3.0; *p* < 0.05; rested *Dbh*^−^, *q* = 5.2; *p* < 0.01: and CFS *Dbh*^−^, *q* = 4.7; *p* < 0.05 (Fig. 6*B*,*D*). Aβ_42_ colocalized with microglial cells labeled with Iba-1; however, there were no differences in Iba-1 percent area in CA1 across the four groups for an overall interaction, *F*_(1,35)_ = 0.0, N.S.; main genotype, *F*_(1,35)_ = 0.2, N.S.; or main sleep effect, *F*_(1,35)_ = 0.5, N.S. An upregulation of CD68 suggests lysosomal activation in microglia and may be a more sensitive measure of microglial activation than Iba-1; thus, we next examined sleep and genotype effects on CA1 CD68. CD68 largely associated with microglia (Fig. 6*B*), and there were main sleep and genotype effects (*F*_(1,28)_ = 7; *p* < 0.05 and *F*_(1,28)_ = 19; *p* < 0.000) and a sleep × genotype interaction (*F*_(1,28)_ = 8; *p* < 0.05). In *Dbh*^+^ mice, CD68 increased in response to CFS (*q* = 5.3; *p* < 0.01), while levels were elevated in rested *Dbh*^−^ mice relative to rested *Dbh*^+^ mice (*q* = 7.1; *p* < 0.001) and did not increase further in response to CFS (*q* = 0.2, N.S.), as summarized in Figure 6*F*. In summary, as with the AEP-APP response in CA1, the CFS increase in CA1 Aβ_42_ is dependent on NE. In contrast, transgenic absence of NE predisposes the mice to increased p-tau in CA1 and EC, in parallel with increased AEP-tau and microglial lysosomal activation. Collectively, these findings support the concept that forebrain balance of Aβ_42_ and p-tau is determined by both NE presence and sleep consolidation.

## Discussion

Two general mechanisms have been proposed by which sleep disruption can perturb brain amyloid and tau homeostasis: increased production and/or release of Aβ and tau from wake-induced neuronal activation and reduced glymphatic clearance (Roh et al., 2012; Xie et al., 2013; Holth et al., 2019). Yet molecular mechanisms underlying sleep-related changes in production, release, and clearance are poorly understood, and little is known of why LCn are vulnerable in both sleep disruption and in AD. Here, we demonstrate that sleep disruption in the form of CFS induces LCn AEP activation with resultant production of AEP-cleaved APP and tau fragments in LCn, fragments known to be neurotoxic and contributory to AD pathology. We also show that NE is required for this CFS-induced AEP activation in the LC and for CFS loss of LCn. AEP activation by CFS was also evident in two AD-vulnerable forebrain regions, CA1 HC and the EC. In these regions, NE played very different roles for each of the two studied AEP targets, tau and APP, where NE deficiency increased AEP-tau and p-tau in a sleep-independent manner, while NE deficiency prevented the sleep-dependent (CFS) increase in AEP-APP and Aβ, demonstrating that NE critically determines the balance of Aβ and tau in AD-vulnerable brain regions across rested and sleep disruption conditions.

LCn activation is important for arousal, and *Dbh*^−^ mice show wake impairments at baseline (less wake time/24 h; Ouyang et al., 2004; Carter et al., 2010). Thus, one possibility for reduced injury in *Dbh*^−^ mice could be reduced sleep fragmentation in *Dbh*^−^ mice in response to CFS. However, in response to CFS, the two strains showed similar arousal indices, as well as similar numbers and lengths of NREM sleep bouts, supporting comparable fragmentation in the two strains. Only *Dbh*^+^ mice evidenced reduced wakefulness/24 h in response to CFS. An impaired homeostatic response to sleep loss has been shown after LCn chemical lesioning (Cirelli et al., 2005), which may explain why CFS did not increase sleep drive in *Dbh*^−^ mice.

The observation that sleep disruption activates AEP in LCn has important implications. AEP activation promotes p-tau, including phosphorylation at Ser202/Thr205, and in the P301S mouse model of tauopathy, absence of AEP prevents mutant tau-mediated CA1 synapse loss and cognitive dysfunction (Basurto-Islas et al., 2013; Z. Zhang et al., 2014). APP is also a substrate of AEP, and in mouse models of AD, deletion or inhibition of AEP normalizes synaptic function, improves cognitive function, and lessens Aβ and tau content and amyloid plaque accumulation (Zhang et al., 2015; Qian et al., 2024). Thus, AEP activation plays essential roles in neuronal injury and dysfunction in diverse AD mouse models, and here we show that CFS can activate AEP in key AD-vulnerable brain regions in the absence of AD genetic mutations and that this activation is dependent on NE. We suggest that the role of AEP in LCn and the forebrain in sleep loss effects on AD should now be examined for it roles in CSD and AD pathology and cognitive impairment.

Discordant AEP activation responses were observed in the forebrain for CFS-induced AEP cleavage of tau and APP. The distinct responses may in part be attributed to differences in subcellular localizations of these two AEP target proteins. APP is rapidly glycosylated and transported anterogradely within the trans-Golgi compartment directly to synapses, where APP positions as a transmembrane protein at synapses (Ferreira et al., 1993). APP is also identified in lysosomes and endosomes, presumably following endocytosis of synaptic membrane fragments with APP. Pro-AEP (the inactive precursor) translocates from the endoplasmic reticulum to lysosomes under conditions of cellular stress. The low pH in lysosomes converts pro-AEP to AEP, which then cleaves APP (Basurto-Islas et al., 2013). Activated AEP may also cleave synaptic transmembrane APP in the ectodomain to facilitate β-secretase cleavage to form Aβ_42_ (Zhang et al., 2015). As the observed CFS-induced AEP-APP response was NE dependent, AEP-cleaved APP may originate in LCn synapses and varicosities within the hippocampus. In support, imaging revealed a punctate pattern for AEP-APP around CA1 neurons in the hippocampus, consistent with a synaptic source, but confirmation of subcellular localization will require electron microscopy or super-resolution microscopy. In contrast with APP's synaptic localization, tau normally localizes within axons as a microtubule-associated protein but can localize to the nucleus with age, where tau may function as a structural nucleic acid binding protein (Anton-Fernandez et al., 2023). Upon metabolic stress and phosphorylation, tau can migrate to the cell body and into dendrites (Ittner et al., 2010). In response to CFS in *Dbh*^+^ mice, we observed AEP-tau within nuclei, somata, and dendrites of CA1 and EC neurons, supporting the concept that CFS results in metabolic stress in CA1 and EC pyramidal neurons, which in turn results in tau mislocalization and increased vulnerability to cleavage by AEP. The increase in p-tau in both the HC and EC in *Dbh*^−^ mice was initially surprising but immediate early gene FosB is elevated in *Dbh*^−^ mice, which upregulates cyclin-dependent kinase-5 (Chen et al., 2000), a key source of neuronal p-tau.

Molecular mechanisms of CSD injury to LCn are not well understood, yet the present work unveils a key driver. CSD imparts metabolic stress in LCn (J. Zhang et al., 2014), and in most experimental paradigms (including short sleep and CFS), CSD results in LCn loss (Shaffery et al., 2012; J. Zhang et al., 2014; Zhu et al., 2015; Deurveilher et al., 2021; Owen et al., 2021). LCn show increased firing frequencies across wake, particularly during exposures to novelty, relative to sleep, and show large bursts in firing rates upon arousals when autoreceptor tone is low in sleep (Aston-Jones and Bloom, 1981). Thus, short sleep and CFS are expected to increase NE release, reuptake, and metabolism in LCn. Here, we demonstrate that NE is necessary for CFS LCn loss. There are at least two mechanisms by which NE may contribute to LCn demise. The first is through AEP activation after NE is metabolized by monoamine oxidase-A to DOPEGAL (S. S. Kang et al., 2020, 2022). AEP processing of tau and APP to toxic fragments may then contribute to neuronal demise. In support of NE-dependent AEP activation, CFS increased both AEP-tau and AEP-APP in LCn in *Dbh*^+^ but not *Dbh*^−^ mice. A second means by which NE may induce toxicity in LCn is through a monoamine oxidase-A–independent increase in oxidized quinones and reactive semiquinones (additional metabolites of NE), which may then promote depletion of glutathione and perturb functions of macromolecules that are critical to cell survival (Napolitano et al., 2011). NE-metabolized, quinone derivatives can also directly activate AEP (Santa-Maria et al., 2004; Bolton and Dunlap, 2017). Because NE serves numerous protective and adaptive roles in the brain, including roles in optimization of vascular, glial, and synaptic responses to increased neuronal activation (O’Donnell et al., 2012), and NE is necessary for appropriate sustained attention, mood, memory and stress responses (Aston-Jones and Bloom, 1981; Thomas and Palmiter, 1997; Sara, 2009; Gompf et al., 2010) and to regulate microglial homeostatic responses to locally increased neuronal activity (Liu et al., 2019; Stowell et al., 2019), and because increased NE within LCn appears deleterious to LCn, NE reuptake, and/or AEP inhibition, rather than NE receptor antagonism should be better options to explore as potential means to prevent CSD and AD neural injury.

In conclusion, NE plays a critical role in the LCn loss observed in response to CFS; NE is essential for CFS AEP upregulation and activation in LCn and the formation of toxic APP and tau fragments and the phosphorylation of tau in LCn, and NE is required for CFS activation of AEP in the HC and EC, as measured with AEP-cleaved APP and increased Aβ_42_ in the HC. By identifying roles for LCn and NE in CFS forebrain Aβ dyshomeostasis, the findings provide both molecular and neuronal subtype windows into how sleep loss may influence AD pathogenesis.
